# Alpine Ski Coaches’ and Athletes’ Perceptions of Factors Influencing Adaptation to Stress in the Classroom and on the Slopes

**DOI:** 10.3389/fpsyg.2019.01641

**Published:** 2019-07-30

**Authors:** Paul Davis, Anton Halvarsson, Wictor Lundström, Carolina Lundqvist

**Affiliations:** ^1^Department of Psychology, Umeå University, Umeå, Sweden; ^2^Department of Behavioural Sciences and Learning, Linköping University, Linköping, Sweden

**Keywords:** emotions, coping, mixed methods, dual career athletes, student athlete

## Abstract

Research examining the student-athlete experience proposes a number of factors that can be both sources of stress and/or support. The dual career pathway offers a number of potential positive outcomes including psychological, social, and financial benefits; however, challenges including time management, fatigue, and restricted social activities are well documented. In consideration of the multidimensional student-athlete experience and the numerous factors that influence the complexity of potential stress, a mixed methods research study design was used in the study. First, data collected from surveys completed by 173 elite junior alpine skiers were analyzed to identify the degree to which athletes report experiencing stress associated with specific aspects pertaining to training, life, and organizational factors. These factors were then explored through semi-structured interviews with six coaches at the associated national elite sport schools. Taken collectively, athletes’ reports of psychophysiological training stress on the Multidimensional Training Distress Scale were low. Scores on the college student-athletes’ life stress scale revealed very low levels of general life stress; although the subscales associated with “performance demand” and “academic requirements” scored marginally higher. Scores on the Organizational Stressor Indicator for Sport Performers indicated low levels of organizational stress. The interviews with coaches elucidated the underlying factors potentially influencing athletes’ positive adaptations to stress as they reported programming a number of strategies to reduce negative outcomes. Coaches aimed to teach athletes self-awareness and regulation strategies through the use of the training diaries and ongoing communication to promote positive adaptation to stress. A number of coaches also worked with sport psychology consultants to optimize athletes’ training and study situations. Traditionally, research has noted high levels of stress in student-athletes due to co-occurring demands (school & sport); however, the data in the present study suggests that optimizing support mechanisms across domains can promote positive adaptations to potential sources of stress.

## Introduction

Research examining the experience of adolescent student-athletes proposes that individuals aiming to achieve high levels of success within both sport and academic domains are prone to increased stress as a result of combined performance demands (Stambulova and Wylleman, 2015; Kristiansen, 2017). The perceived pressure of striving within the intertwined domains has been shown to contribute to reports of symptoms associated with burnout and mental health issues (Sorkkila et al., 2017b, 2018a). An athlete’s ability to adapt to the stressful situation of the dual career pathway (i.e., combining sport and education, sport and work) may be influenced by the contributing factors and support mechanisms that comprise their environment (Stambulova et al., 2015). In particular, within the student-athlete’s environment coaches have been identified as both a potential source of pressure as well as a support (Ronkainen et al., 2018). Further, the role of the coach is to promote athletes’ positive adaptations to the demands of training and competition with the aim of maximizing performance outcomes; their perspective of athletes’ development within the dual career pathway is particularly relevant (Gledhill and Harwood, 2015). Therefore, the purpose of the present study was to examine Swedish elite alpine skiers’ reports of academic and sport related psychological stress in combination with coaches’ perspectives of related factors influencing athletes’ adaptations to stress.

Extensive research investigating the dual career pathway has highlighted problematic aspects derived from the combination of high level sport competition and academic study (see Stambulova and Wylleman, 2015 for a review). Historically, the responsibility for determining how best to navigate the co-occurring demands of academics and sport largely fell upon the athlete (Kristiansen, 2017). In the 1970s the Swedish Sports Confederation acknowledged both the risks and benefits for athletes pursuing a dual career pathway, and identified the need for student-athletes to be able to train and study in an educational environment that could support their sporting aspirations as well as develop academic skills. Subsequently, the first National Elite Sport Schools were established to support athletes in optimizing their holistic health and high performance (Stambulova et al., 2015). Currently, there are 51 National Elite Sport Schools across Sweden supporting about 1200 athletes competing in 30 sports^1^ ; 11 elite sport schools admit athletes competing in alpine skiing. The role of the sport schools in the development of adolescent student-athletes is particularly important as they are generally attended by individuals 16–19 years old; this age group of athletes are typically approaching or commencing the transition from junior to senior competition, and/or considering the difficult decision of withdrawing from participation in elite level competition (Pummell et al., 2008; Stambulova and Wylleman, 2014). The implications of this transition phase can be particularly impactful upon an athlete’s identity as well as his/her adaptation to the stress underlying these formative years in adolescence (Brewer et al., 1993; López et al., 2015b; Gustafsson et al., 2018).

Adolescence has been identified as a key stage in which strategies promoting positive adaptation to stress and resilience can be developed (Gerber et al., 2013; White and Bennie, 2015). Extensive empirical research in sport suggests that athletes displaying high levels of sporting success and general wellbeing are those that can adapt when confronted with adversities and stressors (Fletcher and Sarkar, 2013; Galli and Gonzalez, 2014; Drew and Matthews, 2018). Multiple decades of research activity has sought to identify potential stressors within the athlete environment (Scanlan et al., 1991; Woodman and Hardy, 2001; McKay et al., 2008). Taken collectively, the findings across this research has led to the categorization of stressors into those that are associated with competitive performance, organizational factors, and personal life beyond the sport context (Fletcher et al., 2006; Sarkar and Fletcher, 2013). Adolescent student-athletes in particular, face an intensification of these potential stressors across domains as they enter a phase of increasing elite competition as well as escalating academic demands (Stambulova et al., 2015; Kristiansen, 2017). Moreover, their strategies to adapt to these stressors are in development as they gain increasing experience and feedback of their efficacy (Holt et al., 2005; Galli and Vealey, 2008; White and Bennie, 2015).

A number of psychological protective factors against the negative effects of stress have also been identified (Sarkar and Fletcher, 2013). In particular, the social support an athlete perceives is identified as being particularly influential to stress adaptation (Rosenfeld et al., 1989; Kerdijk et al., 2016; Hagiwara et al., 2017). Teammates, family, and coaches have been identified as being sources of social support for young elite athletes when dealing with competition and organizational stress (Kristiansen and Roberts, 2010). Coaches in particular can influence athletes’ stress appraisals and adaptation, as well as performance outcomes and general health (Arnold et al., 2017; Dixon et al., 2017; Davis et al., 2018). Specifically, the quality of the coach-athlete relationship has been observed to be associated with stress appraisals and athlete wellbeing (Davis and Jowett, 2014; Nicholls et al., 2016). Coaches can promote athletes’ development of resilience and positive adaptation to stress through the provision of social support as well as facilitating the development of emotion regulation strategies and functional skills to cope with stressors (Turner and Jones, 2014; Davis and Davis, 2016; Lu et al., 2016).

Typically high performance coaches operate within either a club setting or national team program separate from the sport school (Ronkainen et al., 2018). However, in Swedish National Elite Sport High Schools coaches are often hired as staff at the school and have the opportunity to act as a link for athletes between the domains of sport and academics (Stambulova et al., 2015). The opportunity for coaches to gain greater proximity to athletes’ academic setting has the potential to increase coaches’ awareness of the holistic experience of student-athletes; subsequently, this knowledge may influence both their coaching behaviors as well as the quality of their relationship with the athletes. Ultimately, this may also optimize student-athletes’ stress adaptation and promote both high performance and health.

In consideration of the significant role of coaches in student-athletes’ stress adaptation, the central research question in the present study aimed to explore the underlying factors that influence student-athletes’ experience of academic and sport related stress. In particular, to gain a comprehensive understanding of the context shaping student-athletes’ reports of academic and sport related psychological stress, qualitative interviews with coaches were compared with athletes’ scores on validated multidimensional stress scales using a mixed methods research design.

## Materials and Methods

Extensive research investigating the dual career pathway in sport has previously used research designs that predominantly focus solely upon student-athletes’ perceptions of a range of variables associated with health and performance (see Stambulova and Wylleman, 2019). Although both qualitative and quantitative methods have been used widely across the study of dual career athletes, very limited studies have used mixed methods research (MMR; e.g., Sorkkila et al., 2018b) and to our knowledge no studies have combined data collected from a variety of participants (e.g., athletes and coaches). In consideration of the present study’s research question, as well as the multidimensionality of student-athletes’ experience of stress and the role of significant others (i.e., coaches) potentially impacting upon the complexity of their stress, a MMR study design was used. Bryman et al. (2008) highlight the following criteria in judging the quality of a study and the decision to use MMR: relevance to research questions; transparency; a rationale for using mixed methods research; and the need for integration of mixed methods findings. Specific to the domain of sport, the use of MMR offers many benefits for sport psychology research (Moran et al., 2011) including the ability to offset weaknesses and provide stronger inferences, triangulation, and completeness (Hagger and Chatzisarantis, 2011; Horn, 2011). Although, Sparkes (2015) and Smith and McGannon (2018) note potential challenges with the use of MMR in sport and exercise psychology research (e.g., problematic assumptions and integrating findings), if these issues are considered throughout the research process the integration of research techniques may provide a thorough investigation of a phenomenon of interest (Teddlie and Tashakkori, 2012). These techniques can be typified by both focused descriptive cross-sectional data collection to identify relationships between multiple factors (Gratton and Jones, 2010), in combination with follow-up approaches aiming to collect rich, descriptive data depicting complex experiences and perspectives (Silverman, 2006). The present study collected quantitative survey responses from student-athletes to analyze their experience of psychological stress, in combination with semi-structured interviews devised by the researchers to explore coaches’ perceptions of athletes’ experiences and associated factors (Kvale and Brinkmann, 2009). The findings from the quantitative and qualitative data were subsequently integrated through a process of comparing and contrasting analyses to provide a more comprehensive understanding of stress adaptation within the dual career pathway of adolescent alpine athletes (Creswell and Tashakkori, 2007).

### Participants

A sample of 173 junior alpine skiers (78 male, 93 female, 2 did not respond) was recruited from the eleven national elite sports high schools in Sweden that admit alpine skiers. The athletes’ mean age was 17.5 (*SD* = 1.15); they reported an average skiing experience of 12.78 years (*SD* = 2.89) and trained on average 13.42 h per week (*SD* = 4.07). The athletes were at various stages of their studies: 58 (34%) first, 51 (29%) second, 38 (22%) third, and 26 (15%) final year of study.

To explore factors related to the student-athletes’ experiences of psychological stress a purposive sampling technique was adopted (Flick, 2008) to recruit a sample of six coaches (five male, one female), that were each employed at a different national sport high school from which the alpine skiers were recruited. The mean age of the coaches was 46.25 (*SD* = 6.55); they reported being involved in coaching alpine skiing an average of 14.00 years (*SD* = 2.71). The present study was appraised and approved by Umeå University’s Coaching Program review panel for student research, the panel is responsible for evaluating ethical aspects of the research; additional ethical scrutiny was deemed not to be required as per applicable institutional and national guidelines and regulations. Principals at each of the participating national elite sports high schools reviewed the ethical considerations of the study prior to approving the commencement of data collection.

### Measures

#### Multi-Component Training Distress Scale

The Multi-component training distress scale (MTDS; Main and Grove, 2009) is a multidimensional questionnaire consisting of 22 items that aim to assess symptoms concerning training distress. It includes six subscales in total; four subscales are measured in terms of their frequency: “depressed mood,” “perceived vigor,” “perceived stress” and “general fatigue” scored on a five-point Likert scale ranging from “*never*” (0) to “*very often*” (4). The subscale perceived vigor is scored positively, with higher scores reflecting greater frequency of experiencing higher levels of energy. The remaining two subscales are measured in terms of their intensity: “physical symptoms,” “sleep disturbance” scored on a five-point Likert scale ranging from “*not at all*” (0) to “*extreme amount*” (4). The MTDS has acceptable reliability and evidence of construct validity; in the present study the overall internal consistency was α = 0.84. More specifically, the individual subscales demonstrated the following Cronbach’s α: depressed mood = 0.84, perceived vigor = 0.76, physical symptoms = 0.50, sleep disturbances = 0.87, perceived stress = 0.81, fatigue = 0.80.

#### College Student-Athletes’ Life Stress Scale

The college student-athletes’ life stress scale (CSALSS; Lu et al., 2012) is designed to capture the current state of stress among intercollegiate student-athletes. It is comprised of 24 items that reflect potential stressors respondents may encounter in their everyday life and in sports. The scale includes eight subscales divided into two categories; sport-specific stressors (i.e., “sports injury,” “performance demand,” “coach relationships,” “training adaptation”) and general life stressors (i.e., “interpersonal relationship,” “romantic relationship,” “family relationship,” “academic requirements”). The CSALSS presents potential answers in terms of their frequency scored on a six-point Likert scale ranging from 1 (*never*) to 6 (*always*). The CSALSS examines sport-specific stressors and general life stressors and has reported Cronbach’s α of its eight factors ranging from 0.72 to 0.86, with the reliability for all items being 0.88. The scale has been subject to further tests for discriminant and concurrent validity and report standard psychometric results (Lu et al., 2012). The CSALSS demonstrated good internal consistency with Cronbach’s α = 0.82 for sport-specific stress and 0.83 for general life stress in the present study. More specifically, the individual subscales demonstrated the following Cronbach’s α: sports injury = 0.82, performance demands = 0.60, coach relationships = 0.80, training adaptation = 0.67, interpersonal relationships = 0.86, romantic relationships = 0.62, family relationships = 0.81, academic requirements = 0.72.

#### Organizational Stressor Indicator for Sport Performers

The Organizational Stressor Indicator for Sport Performers (OSI-SP; Arnold et al., 2013) was developed to comprehensively measure the organizational pressures that sport performers may encounter. The OSI-SP consists of 23 items with five subscales: “goals and development,” “logistics and operations,” “team and culture,” “coaching,” and “selection.” Each of the subscales are evaluated in terms of their frequency, intensity, and duration. Items on the OSI-SP are scored on a six-point Likert scale ranging from 0 (*never*) to 5 (*always*). The OSI-SP has been validated through a series of studies, with support provided for its internal consistency (Cronbach’s alpha coefficients ranged from 0.75 to 0.85 for the frequency scales, 0.71 to 0.83 for the intensity scales, and 0.74 to 0.83 for the duration scales), content, concurrent, discriminant, and factorial validity (Arnold et al., 2013). In the present study the frequency aspect of the dimensions were measured; Arnold et al. (2013) state that researchers requiring a shorter version of the indicator would benefit using the frequency scale alone. Regarding internal consistency, in the present study the five subscales of OSI-SP demonstrated the following Cronbach’s α: goals and development = 0.81, logistics and operation = 0.89, team and culture = 0.86, coaching = 0.89, selection = 0.43. In consideration of the low Cronbach’s α for the “selection” subscale we inspected the two items comprising the subscale (i.e., “*how my team is selected*” and “*selection of my team for competition*”) and determined that they were not entirely relevant for these athletes as their selection to the team was established upon admission to the high school; therefore the subscale was not included in subsequent analyses.

The process of back-translation (Brislin, 1970) was used to translate all of original questionnaires from English into Swedish. Specifically, a bilingual individual first translated the English version of each scale into Swedish and then another bilingual individual independently translated the Swedish version back to English to compare it with the original versions for confirmation of clarity and accuracy. Revisions to the Swedish versions were discussed and final versions were agreed following the process of inter-translator reliability.

### Procedure

Initial discussions and a subsequent study proposal outlining the purpose of the present study were exchanged between the authors of the study and the Swedish Ski Association. Upon receiving an indication of support from the Ski Association, coaches at all of the National Elite Sports High Schools were verbally informed about the purpose of the study at the Swedish National Junior Championships. Emails were then sent to principals and head coaches at each school to update and inform them about the protocol of the study. All 11 schools replied by phone or email with approval and contact information of an individual at the school to support data collection; the total number of student-athletes at the schools with the potential to participate was 296, subsequently this number of questionnaires was sent across the schools.

The participating athletes completed the questionnaire in a classroom setting or a training session under the supervision of a teacher or coach. Prior to completing the questionnaire, athletes were provided information sheets about the nature of the study. They were informed participation was voluntary and that they could skip any questions they did not want to answer or discontinue their participation at any time. After the participants had signed the consent form and completed the questionnaires they placed their questionnaires and consent form in two separate envelopes to ensure their completed responses were kept separate from their names and any potentially identifying information. The sealed envelopes were then mailed to the research team. The data collection occurred toward the end of the competitive season with approximately 6 weeks of events remaining.

Following the analysis of the questionnaire data, it was determined that greater understanding of the alpine athletes’ dual-career context was required and that qualitative data collected from an alternative source (i.e., coaches) would be relevant in exploring the research question as well as support the rationale for using MMR (Bryman et al., 2008). The qualitative interviews were developed upon reflection of the findings from the athletes’ responses and in review of research literature of the student-athlete experience. More specifically, coaches’ perceptions of the potential sources of stress and the support mechanisms that were present within the context of the National Elite Sports High Schools were explored. Therefore, the questions comprising the interview guide were grouped into three sections: (i) perceptions of stress as it relates to student-athlete performance and wellbeing; (ii) sources of stress relating to performance and life in general; (iii) strategies/support systems targeting student-athlete stress. The interviews were semi-structured and contained primarily open-ended questions aimed at encouraging insightful responses. Initially, introductory questions were asked allowing the participant to discuss their general perceptions/personal definition of psychological stress. Questions progressed to be more specific in nature by asking participants to outline the implications of psychological stress on student-athletes’ performance and wellbeing, contributing factors, and sources of support.^2^

Coaches at the schools where athletes completed the questionnaires were contacted via email and/or telephone and provided information about the research plan to conduct interviews to follow up the questionnaires and were asked if they would be interest in taking part in the study. Those who responded with a confirmation of intention to participate were subsequently contacted to arrange for the completion of the interview. The second author conducted all the interviews as he possesses experience of alpine ski coaching and was able to share experiences from the sport to develop rapport with the coaches.

Prior to commencing the interview, each participant provided his/her consent to be recorded and was reminded that participation in the study was voluntary. The confidentiality of the coaches was assured by the author conducting the interviews. The interviews took place over a 2-week period and were conducted individually over the telephone. The participants were told they would be interviewed about their perceptions of student-athletes’ experiences of stress related to school and performance, as well as potential sources of stress and support. When required, follow up questions were used to encourage further discussion or if participants did not fully understand the question. The interviews ranged in time from 15 to 35 min and were simultaneously recorded on two digital audio recorders. The interviews were conducted in Swedish and subsequently transcribed verbatim; transcripts were checked by the interviewer to ensure accuracy.

### Data Analysis

First, to examine student-athletes’ experience of psychological stress, responses to questionnaires were analyzed to identify potential factors that were highlighted by the athletes. Descriptive statistics and bivariate correlations are reported to identify the degree to which athletes reported experiencing stress associated with these factors. In conjunction with the quantitative data analyses of athletes’ reports, coaches’ perceptions of the factors influencing student-athletes’ psychological stress were analyzed. The transcripts from the interviews with the coaches underwent coding and were scrutinized using inductive content analysis.

#### Coding

A variety of approaches to analyzing qualitative data has been used within sport psychology research exploring psychological stress; in the present study, a conventional content analysis procedure was selected for use to analyze, organize, and articulate the responses of participants. Inductive content analysis was used to assist with the development and interpretation of categories from each of the interview transcripts (Hsieh and Shannon, 2005). This technique was identified as being applicable as it permitted perceptions of specific factors associated with sources of stress to be acknowledged (e.g., organizational stress); categorizing the coaches’ responses also helped differentiate the multidimensional aspects of the dual-career pathway (e.g., academic and sport demands).

The initial stage of the coding process was open coding, this allows researchers to engage the data in the transcripts and promote the identification of raw-data quotes related to student-athletes’ psychological stress (Hsieh and Shannon, 2005). The first and second authors read through the transcripts independently; they produced notes to each segment to facilitate subsequent reviews and enable the coding of chunks of data regardless of the context. Quotes which characterized common themes were collated and labeled as categories prior to being pooled and identified as higher order categories (Aronson, 1995). In sum, categories were structured into general dimensions promoting the development of a comprehensive overview of participants’ collective perceptions to be established (Patton, 2002; Vaismoradi et al., 2016). Two members of the research team undertook the process of coding and established the higher order themes independently; these themes were debated extensively and agreed upon amongst the research team. Further, colleagues of the investigators acted as critical friends (Sparkes and Smith, 2014) and offered a critical perspective of the proposed themes that were identified from the analysis process.

## Results

The present study sought to examine the psychological stress of student-athletes studying at National Elite Sport High Schools in Sweden and competing in alpine skiing; underlying factors associated with athletes’ adaptation to stress were also explored. First, data collected from a survey were analyzed to identify the degree to which athletes reported experiencing stress associated with specific aspects pertaining to training, life, and organizational factors. These factors were then explored through semi-structured interviews undertaken with coaches at the high schools to elucidate their influence upon stress adaptation within the dual career pathway of adolescent alpine athletes.

To examine the extent to which athletes reported symptoms of psychophysiological stress related to training, scores on the MTDS were scrutinized. Taken collectively, athletes’ reports of training distress were generally low (see Table 1, for descriptives) with most of the subscales’ (i.e., depressed mood, physical symptoms, sleep disturbance, perceived stress) mean score positioned between the range of “*a little bit*” and “*moderate amount*;” the only exception was general fatigue (*M* = 2.44; *SD* = 0.82) scoring between “*moderate amount*” and “*quite a bit*.” Athletes also reported higher scores on the positively oriented subscale perceived vigor (*M* = 2.51; *SD* = 0.68) indicting they frequently experienced higher levels of energy and feeling alert.

**TABLE 1:** Descriptive statistics, correlations for multi-component training distress scale, college student-athlete life stress scale, and organizational stressor indicator for sport performers.

	**1**	**2**	**3**	**4**	**5**	**6**	**7**	**8**	**9**	**10**	**11**	**12**	**13**	**14**	**15**	**16**	**17**	**18**
1. Stress	1																	
2. Depressed mood	0.591^∗∗^	1																
3. Vigor	−0.188^*^	–0.270^∗∗^	1															
4. Physical symptoms	0.204^∗∗^	0.265^∗∗^	0.065	1														
5. Sleep disturbance	0.459^∗∗^	0.555^∗∗^	−0.173^*^	0.289^∗∗^	1													
6. Fatigue	0.457^∗∗^	0.434^∗∗^	–0.358^∗∗^	0.242^∗∗^	0.486^∗∗^	1												
7. Injury	0.253^∗∗^	0.408^∗∗^	–0.001	0.365^∗∗^	0.291^∗∗^	0.219^∗∗^	1											
8. Performance demands	0.523^∗∗^	0.532^∗∗^	–0.060	0.335^∗∗^	0.317^∗∗^	0.304^∗∗^	0.423^∗∗^	1										
9. Coach relationship	0.371^*^	0.321^∗∗^	–0.229^∗∗^	0.117	0.174^*^	0.310^∗∗^	0.107	0.386^∗∗^	1									
10. Training adaptation	0.478^∗∗^	0.509^∗∗^	–0.218^∗∗^	0.253^∗∗^	0.303^∗∗^	0.416^∗∗^	0.280^∗∗^	0.532^∗∗^	0.659^∗∗^	1								
11. Interpersonal relationship	0.345^∗∗^	0.548^∗∗^	–0.133	0.165^*^	0.158^*^	0.164^*^	0.216^∗∗^	0.459^∗∗^	0.421^∗∗^	0.486^∗∗^	1							
12. Romantic relationship	0.215^∗∗^	0.447^∗∗^	–0.083	0.131	0.067	0.279^∗∗^	0.216^∗∗^	0.369^∗∗^	0.363^∗∗^	0.451^∗∗^	0.575^∗∗^	1						
13. Family relationship	0.358^∗∗^	0.465^∗∗^	−0.171^*^	0.187^*^	0.291^∗∗^	0.366^∗∗^	0.197^*^	0.390^∗∗^	0.239^∗∗^	0.387^∗∗^	0.412^∗∗^	0.252^∗∗^	1					
14. Academic requirements	0.467^∗∗^	0.408^∗∗^	–0.070	0.227^∗∗^	0.257^∗∗^	0.404^∗∗^	0.220^∗∗^	0.437^∗∗^	0.246^∗∗^	0.403^∗∗^	0.255^∗∗^	0.304^∗∗^	0.382^∗∗^	1				
15. Goals and development	0.544^∗∗^	0.542^∗∗^	–0.108	0.389^∗∗^	0.419^∗∗^	0.319^∗∗^	0.643^∗∗^	0.737^∗∗^	0.362^∗∗^	0.504^∗∗^	0.319^∗∗^	0.250^∗∗^	0.393^∗∗^	0.443^∗∗^	1			
16. Logistics operations	0.492^∗∗^	0.509^∗∗^	–0.064	0.394^∗∗^	0.311^∗∗^	0.397^∗∗^	0.308^∗∗^	0.485^∗∗^	0.439^∗∗^	0.498^∗∗^	0.430^∗∗^	0.460^∗∗^	0.399^∗∗^	0.414^∗∗^	0.549^∗∗^	1		
17. Team and culture	0.507^∗∗^	0.507^∗∗^	−0.156^*^	0.328^∗∗^	0.416^∗∗^	0.246^∗∗^	0.252^∗∗^	0.474^∗∗^	0.481^∗∗^	0.452^∗∗^	0.536^∗∗^	0.264^∗∗^	0.391^∗∗^	0.311^∗∗^	0.479^∗∗^	0.570^∗∗^	1	
18. Coaching	0.382^∗∗^	0.382^∗∗^	–0.251^∗∗^	0.027	0.131	0.283^∗∗^	0.079	0.266^∗∗^	0.812^∗∗^	0.603^∗∗^	0.326^∗∗^	0.277^∗∗^	0.216^∗∗^	0.243^∗∗^	0.306^∗∗^	0.346^∗∗^	0.461^∗∗^	1
M	1.96	1.16	2.51	1.53	1.05	2.44	2.32	2.46	1.86	1.99	1.73	1.64	1.72	2.50	1.74	0.84	1.10	0.78
SD	0.81	0.82	0.68	0.71	1.03	0.82	1.32	0.94	0.96	0.88	0.98	0.81	0.94	0.99	1.02	0.66	1.04	1.07

*N* = 173; ^*^p < 0.05; ^∗∗^*p* < 0.01. Range of scores for: “depressed mood”, “perceived vigor”, “perceived stress” “general fatigue” is 0 to 4 representing frequency; “physical symptoms”, “sleep disturbance” is 0 to 4 representing intensity; “sports injury”, “performance demand”, “coach relationships”, “training adaptation”, “interpersonal relationship”, “romantic relationship”, “family relationship”, “academic requirements” is 1 to 6 representing frequency; “goals and development”, “logistics and operations”, “team and culture”, “coaching” is 0 to 5 representing frequency.

Scores on the MTDS were then compared with measures of sport specific stressors on the CSALSS (i.e., sports injury, performance demand, coach relationships, training adaptation), again athletes’ reported very low levels of stress with mean scores between “*never*” and “*rarely*” for the factors of coach relationships and training adaptation. The subscales associated with sports injury and performance demand, scored marginally higher with the mean scores between the range of “*rarely*” and “*sometimes*” (see Table 1, for descriptives). General fatigue scores on the MTDS were correlated with measures of sports injury *r* = 0.219, *p* < 0.01 and performance demand *r* = 0.304, *p* < 0.01; although sleep disturbance was noted as being most strongly associated with general fatigue *r* = 0.486, *p* < 0.01. In consideration of athletes’ scores on the OSI-SP, the subscales of goals and development and team and culture were on the low end of the six point range between “*rarely*” and “*sometimes*,” whilst the subscales of logistics and coaching were in the range between “*never*” and “*rarely*” (see Table 1, for descriptives).

Athletes’ reports of general life stressors were also examined using scores on the CSLASS relating to the subscales identified as interpersonal relationship, romantic relationship, family relationship, and academic requirements (see Table 1, for descriptives). Scores indicating the frequency that athletes experienced these stressors were very low with means ranging between “*never*” and “*always*” for all of the subscales except academic requirements (*M* = 2.50; *SD* = 0.99) in the range between “*rarely*” and “*sometimes*.” It may be noted that academic requirements was the highest scoring source of stress of all the measures recorded, and was most strongly correlated with reports of perceived stress on the MTDS *r* = 0.467, *p* < 0.01. Overall, the analyses of athletes’ responses to the various assessments indicated that they perceived relatively low levels of stress.

To gain a comprehensive understanding of the context shaping student-athletes’ reports of academic and sport related psychological stress, qualitative interviews with coaches were undertaken to collect data exploring the conditions influencing athletes’ scores on the stress scales. In particular, the factors influencing athletes’ overall experience of stress were elucidated by the interviews with the coaches as they identified that athletes’ expected themselves to perform to a high level in both sport and academic study (see Table 2 for higher order themes and categories). Further, multiple coaches outlined that athletes’ previous experience of being “high achievers” in sport and academia guided expectations and evaluations of success. “There are many that are high achievers in elementary school and then they come here and want to continue on the same level and have very good results” (P1).

**TABLE 2:** Summary of higher order themes and categories pertaining to coaches’ perceptions of factors influencing athletes’ stress adaptation.

**Higher order themes**	**Categories**	**Subcategories**
**Sources of**	Appraisals and	Athletes’ previous levels of success
**stress**	expectations of	Athletes’ personal sport goals
	performance	Athletes’ comparison with teammates
		Parents’ expectations
	Academic requirements	Conflict between balancing sport and academic demands
		Athletes’ personal standards and goals
	Limited resources	Time management
		Costs of competition
	Friends and Family	Over-involvement
		Challenge of independent living
		Fear of missing out/feeling isolated
**Support**	Friends and Family	Recovery outside of sport
**mechanisms**		Investment in talent development
	Teammates	Empathy
		Affiliation
	Coaches	Monitoring of athletes
		Connection and communication
		Programming of training
	School	Flexibility
		Support staff and services
		Sport focused educational environment

Coaches also differentiated the sources of stress athletes experienced; they noted that the stress had varying implications for performance and wellbeing depending on its initial source. In particular, it was suggested that stress may facilitate sport performance if athletes were able to understand its origin and regulate its intensity; for example, fear arising from the anticipation of a challenging course could enhance an athlete’s concentration and promote greater investment in preparation, “The nervousness itself is a type of additional resource. And it’s something they should learn to manage and use for something positive. You get a focus and you get a certain rise of adrenaline that you can use” (P1).

Alternatively, coaches viewed academic stress as being largely negative for athlete wellbeing. Collectively, the coaches outlined a number of factors contributing to academic stress; in particular, time management was a challenge when the time athlete’s spent traveling and training was difficult to balance with academic requirements. Coach number 3 outlined how the issue of balancing time allocated between sport and studies is exacerbated when athletes are attempting to make the national team and choosing to attend an increased number of competitions in order to gain FIS points:

In our sport there is a stress about being selected for a junior national team and it is of course a great advantage to join a junior team; you get skis, you get clothes, you get everything possible, and a lot of training. It’s obviously a great advantage to be accepted there and it also comes as a stress. You can compete 100 times in 1 year or you can compete 25 and then the question is what is best (strategy to make the team). Plan 25 competitions or leave it up to fate all the time and hope that some time it will work.

The financial cost associated with traveling was also noted by three coaches as a potential stressor for the athlete and their family. Investing in the athletes’ pursuit of elite level sporting success placed a burden on the family in terms of costs as well as time spent traveling to competitions. On a related note, three coaches reported that some parents contribute to athletes’ stress by being too involved. “Committed parents who are behind their children and helping in a healthy way, as I see it, are the ones who are in the background. While those that go ‘all-in’ and plan competitions and are involved in training and everything else, that can be wrong” (P3). However, family and friends at home (outside of the high school) could also provide support to athletes; sincere and non-judgmental interest in the athlete’s development was appreciated by the coaches. Moreover, family and friends may offer an outlet for recovery by providing a space for athletes that was not connected directly with skiing or training. “…it can be nice to spend time with people who don’t ask about how much you squat or how fast you ran 3000 meters ….” (P4). Although, one coach suggested that the potential for feelings associated with a “fear of missing out” can arise when athletes are aware of challenges managing social relationships via connections with friends on social media.

Athletes’ relationships within sport were also influential to their accounts of psychological stress. Multiple coaches provided details about how relationships between teammates were largely positive as the understanding of the shared experience lead to feelings of empathy being exchanged. However, two coaches did note that when athletes compared themselves with teammates’ rates of development, it could be a source of stress, “They should not compare their results but they do it all the time” (P2).

All of the coaches predominantly viewed themselves as being supportive in athletes’ positive adaptations to stress. The coaches reported programming a number of strategies to reduce the negative effects of stress. First, they spoke about the importance of monitoring levels of stress through communication with the athlete. Frequently “checking in” was noted as being central to understanding the athlete experience; although, varying levels of frequency and formality were reported across the coaches’ interviews. Most schools scheduled meetings at least twice an academic term (e.g., beginning and middle), with some schools scheduling meetings to occur monthly or once a week. Three coaches also mentioned less formal opportunities for gaining status updates from athletes when seeing them at the school or time spent together at training, “Usually you can take the lift up with someone to check how it is going” (P2).

Remote forms of monitoring athletes’ adaptation to psychosocial and training factors were also outlined by coaches; training diaries were used at some of the schools. At one school first year students were encouraged to keep a journal of their general thoughts and experiences, then in the second year it was more structured and obligatory. Other schools used online training logs that were shared between athletes and coaches and provided a basis for follow up discussions. “Usually once every week or every second week I will take a look in the training journal to check if they are OK and doing what they are supposed to be doing” (P6).

Through the use of the training diaries and ongoing communication, coaches aimed to teach athletes self-awareness and regulation strategies to promote positive adaptation to stress associated with training and competition demands. Coaches also adopted more pro-active roles in optimizing recovery and preventing negative adaptations, “…many are very, very ambitious and sometimes our role might be to put on the ‘brakes’ more than to apply the ‘gas”’ (P3). The programming of training sessions was often based upon consideration of athletes’ academic requirements; specifically, four coaches outlined limiting training sessions in order to offer time for academic demands. For example, it was a common practice at multiple schools for coaches to avoid training on the Monday following a weekend competition in order for students to catch up on the school work they likely missed on the preceding Friday due to travel arrangements.

The elite sport high schools also attempted to support the student-athletes by offering flexible schedules for classes and assessments that complemented competition schedules. Further, mental health support services were available at all of the schools; however, these provisions were only initiated in severe cases, and coaches suggested that this was not required very often. Two coaches commented that their school had mental coaches that engaged with the athletes to varying degrees. These mental coaches often worked across all the sports offered at the school “I think it is very good, that we have been given the opportunity to have a mental coach; that we can cooperate and help each other” (P2). Although the alpine coaches noted that the mental coach’s effectiveness was enhanced when he/she possessed sport specific knowledge. In terms of more sport specific support, one school offered mentors from the skiing community to the athletes in order to provide an additional support mechanism in preventing negative adaptation to stress. “An athlete should be able to check with their training mentor to check where they are heading, this can help them feel secure” (P3).

## General Discussion

Previous research highlights that student-athletes are typically extremely motivated to excel in both education and sport (Lupo et al., 2015; Stambulova et al., 2015; Ryba et al., 2017) and coaches in the present study suggest that the alpine athletes they work with also expect themselves to be successful in both domains. However, athletes’ scores across the measures of the sport and life stress indicate they were not experiencing particularly high levels of stress. As a source of stress, academic requirements scored highest; although athletes’ reports indicate that stress related to demands at school were not frequently a cause for concern. Research by López et al. (2015a) of Spanish student-athletes highlights that the most significant barrier to studying is time management. Time management was also reported by coaches in the present study as being a challenge for the athletes; therefore, time management skills appear to be an important skill to teach student-athletes at an early stage in the dual career process.

In the present study, coaches outlined that time spent traveling to competitions has implications that extend beyond the athlete to include their family. Research examining stress in youth sport identifies that the time and money spent in high level sport participation can have a negative impact upon the wellness of both the athletes and the parents due to excessive investment (Harwood and Knight, 2009). The alpine student-athletes indicated that their family and romantic relationships were not frequently a source of stress; coaches also outlined how friends outside of sport were a source of support in providing an outlet for alternative interests and recovery. Related research with data collected from sport parents suggests, however, that maintaining these relationships can be difficult for talented young athletes (Elliott et al., 2018). In consideration of the positive effects of friendships outside of sport found in the present study, and the difficulties in maintaining these relationships reported in previous research, it appears important to support athletes’ development of social skills and efforts in nurturing important social connections.

Relationships within sport between teammates were also noted to be influential to athletes’ stress adaptation. Coaches suggested that teammates for the most part were supportive of each other, and empathy associated with shared experiences helped athletes’ stress management. Athletes indicated that the team and culture surrounding them was not a frequent source of stress. Research investigating the availability of social support from teammates has been shown to be associated with lower risk of burnout and enhanced self-determined motivation (DeFreese and Smith, 2013; Appleby et al., 2018). This is particularly important for coaches, sport psychology practitioners, and sport schools to note as research indicates that athletes’ performance expectations in the domains of sport and school can influence their risk for burnout (Sorkkila et al., 2017a, 2018b).

A central finding taken from the integration of the quantitative and qualitative data analysis was the role of the coach in athletes’ stress adaptation and the underlying mechanisms of developing and maintaining a high quality coach-athlete relationships. Athletes’ scores on the measure of coaches as a source of organizational stress were the lowest recorded on the scale. In review of the interviews with the coaches it was apparent that extensive efforts were made on the part of coaches to connect with their athletes through a range of communication strategies. Research suggests that effective communication between a coach and athlete can optimize the coach-athlete relationship and assist in protecting athletes from negative implications of stress (e.g., exhaustion; Rhind and Jowett, 2011; Davis and Davis, 2016; Lu et al., 2016; Davis et al., 2018). In addition to regular face to face communication across a number of venues (e.g., chairlift, school hallway) coaches used remote communication tools to collect information from the athletes. The collection of subjective self-ratings (e.g., stress, mental fatigue, motivation) has been shown to be important information in monitoring athletes’ training load to optimize performance and prevent injury (Coyne et al., 2018). Further, feedback from athletes was integrated with information regarding other factors influencing athletes’ wellbeing (e.g., academic demands), and coaches reported adjusting training protocols accordingly. Sorkkila et al. (2018b) interviews with Finnish athletes highlight that athletes appreciate adaptations to training schedules in consideration of academic demands as well as competitive seasons.

The flexibility of academic and training schedules was one feature of the National Elite Sport Schools that appeared to benefit athletes’ stress adaptation. Further coaches noted the provision of mental health support services and mental skills coaches within the schools acted as support mechanisms for athletes. However, a lack of sport specific knowledge on the part of the psychological support could be a barrier to their effectiveness. To obtain a successful support provision, the importance of applied sport psychology practitioners to thoroughly learn about the sport and its conditions is well documented (e.g., Ravizza, 1988; Gould et al., 1991; Pain and Harwood, 2004). Sport specific support can also be sought through the use of mentors and their effectiveness is well established within other areas of sport (e.g., women coaches; Vinson et al., 2016), and many North American sport programs use mentors extensively with student-athletes (e.g., Perna et al., 1996; Hoffmann and Loughead, 2016); however, their use in European academic-sport programs to promote athletes’ health and high performance has not been as well documented or evaluated.

The present mixed methods research study not only provided an overview of various psychological stress symptoms reported by elite adolescent alpine student-athletes, it also offered unique insight from the coaches responsible for working with the athletes in their sport and academic development. Thus, the results contribute not only to an understanding of various stressors that may be present when adolescents attend national elite sport schools but also various efforts (e.g., support by mentors and practical arrangements) that may facilitate the combination of sport and studies. Importantly, the results in this study indicate that the combination of academic study and sport does not automatically induce high levels of stress among young athletes. Rather, the thoughtful efforts of the coaches to support and care for the athletes and the overall structure and support resources the sports program itself includes seem to be of great importance to young athletes’ stress experiences. A comprehensive understanding of different supportive factors in the environment and personal strategies that young athletes may need to successfully develop both in school and in sport also helps to customize and increase relevant support to this young population. This project is the first to focus specifically on student-athletes competing in the sport of alpine skiing, as well as recruit coaches directly employed at the elite sport high schools.

The study does, however, have a number of limitations that warrant discussion. Specifically, the cross-sectional data does not permit conclusions regarding causation to be determined. Longitudinal research would enhance knowledge derived from the present study and enable researchers to observe changes in stress adaptation and fluctuations of the impact of specific factors over time. Moreover, longitudinal research incorporating objective measures of stress reactivity (e.g., biomarkers; Blume et al., 2018) would supplement self-report data. One challenge to longitudinal data is the attrition of participants; the present study may also have suffered indirectly from this issue as those student-athletes that were potentially overwhelmed with stress may have been absent at the time of data collection and/or dropped out of the school earlier in the course of their studies. That said, the interviews with the coaches offered the opportunity for those students that may have dropped out from the program to be represented from the coaches’ perspectives. The contributing factors influencing student-athlete drop out are difficult to capture with traditional approaches to data collection undertaken at the schools. Future research investigating stress adaptation in sport would be enhanced by collecting data from individuals that choose to withdraw from the stressful situation. Additionally, collecting data from multiple sources (e.g., coaches, parents) that were involved with the athlete’s experience may elucidate the factors influencing stress adaptation.

Extensive research attention has been paid to the dual career pathway and advanced understanding of the student-athlete experience. Further research is required with alternative research designs and methods using a variety of data sources to gain greater insight from key stakeholders. Developing a more comprehensive understanding of the factors underlying stress adaption will enhance both the performance and holistic health of the student-athlete. The National Elite Sport Schools in Sweden were originally implemented to facilitate the combination of school and high-level sport. Thus, the aim was thereby to contribute to a positive and helpful overall solution for young athletes who wished to strive to reach the elite sport level in their sports. Contrary to this original aim, a traditional assumption in sport psychology research is that the combination of academic study and high-level sport may also involve challenges that could make athletes vulnerable to increased levels of stress (cf. Stambulova and Wylleman, 2015; Sallen et al., 2018). In the present study, the stress levels reported by the athletes were nevertheless relatively low in intensity. Thus, the data in the present study do not support the assumption of increased stress levels among student-athletes. Importantly, what has been less considered in previous research is the consideration of various levels of psychosocial resources student-athletes possess, their level of resilience, as well as the possibility of increased maturity and personal development that attending a national elite sport school may also plausibly contribute. In light of this consideration, instead of a unilateral search of reported stress experiences based on common assumptions, which may also increase the risk of confirmation-bias, future research would be well served by paying attention to potential psychosocial resources and supportive systems within the school-sport environment which may act to counteract stress and potentially promote psychological growth among student-athletes. The present study contributes to the literature with a first step toward such an approach in which the focus was to explore both reported stress experiences, as well as contextually supportive factors in the student-athlete life-situation.

## Data Availability

The datasets generated for this study are available on request to the corresponding author.

## Ethics Statement

This study was carried out in accordance with the recommendations of “Tränarprogrammet/Coaching Program review committee for Student Research at Umeå University” with written informed consent from all subjects. All subjects gave written informed consent in accordance with the Declaration of Helsinki. The protocol was approved by the Tränarprogrammet/Coaching Program review committee for Student Research at Umeå University as well as each of the participating schools.

## Author Contributions

PD designed the study, analyzed the data, and prepared the manuscript. AH designed the study, collected and analyzed the data, and reviewed the manuscript. WL designed the study, collected and analyzed the data. CL designed aspects of the study, consulted on the analyses and wrote sections as well as reviewed the manuscript.

## Conflict of Interest Statement

The authors declare that the research was conducted in the absence of any commercial or financial relationships that could be construed as a potential conflict of interest.

## Footnotes

1 http://www.svenskidrott.se/elitidrottpagymnasiet/

2 The interview guides are available from the first author upon request.
